# Treatment outcome of tuberculosis among Human Immunodeficiency Virus positive patients in Eastern Ethiopia: a retrospective study

**DOI:** 10.11604/pamj.2018.30.32.12554

**Published:** 2018-05-16

**Authors:** Fitsum Weldegebreal, Habtamu Mitiku, Zelalem Teklemariam

**Affiliations:** 1 Haramaya University, College of Health and Medical Sciences, Harar, Ethiopia

**Keywords:** Tuberculosis, treatment success, transferred out, died, defaulter, failure, Human Immunodeficiency Virus, Eastern Ethiopia

## Abstract

**Introduction:**

Tuberculosis is the leading cause of morbidity and mortality among people living with Human Immunodeficiency Virus/Acquired Immunodeficiency Syndrome worldwide. Although Human Immunodeficiency Virus related tuberculosis is both treatable and preventable, incidence rates continue to climb in developing countries where both infections are endemic. The aim of this study was to assess the treatment outcome of tuberculosis among Human Immunodeficiency Virus positive patients attending in three hospitals of Eastern Ethiopia.

**Methods:**

A retrospective clinical record review was conducted for 627 Tuberculosis and Human immunodeficiency virus co-infected patients registered from January 2008 to January 2014 cards were reviewed in three hospitals of tuberculosis clinics of Eastern Ethiopia from December 2015 to February 2016. The three hospitals were selected based on their high patient load of TB-HIV co infection and the presence of ART and TB units. Data on patient's details and tuberculosis treatment outcome were collected using standardized report format of National Tuberculosis and Leprosy Control Programme (NTLCP). The collected data were analyzed by Statistical Package for Social Sciences (SPSS) software Version 16.

**Results:**

The overall treatment success rate was 78.3%. Of the total TB-HIV co infected study participants, 17.9% cured, 60.4% treatment completed, 8.6% died, 0.6% failure, 1.8% defaulter and 10.7% transferred out. Those participants in the age groups of less than or equals to 18 years old (Adjusted Odds Ratio = 1.990, 95% Confidence Interval: 1.01, 3.350), extra pulmonary tuberculosis (Adjusted Odds Ratio = 1.51, 95% Confidence Interval = 1.12, 3.42), on antiretro viral therapy (Adjusted Odds Ratio = 1.54, 95% Confidence Interval = 1.252, 3.910) were more likely to have higher treatment outcome than each of the above variables counter parts.

**Conclusion:**

The rate of treatment success in this study was lower than recommended rate by World Health Organization. Thus this study recommends improving counseling during tuberculosis treatment, providing home visits and motivation of patients, improving defaulter tracing and health information dissemination in order to reduce treatment interruption.

## Introduction

Tuberculosis (TB) is the leading cause of morbidity and mortality among People Living With Human Immunodeficiency Virus/Acquired Immunodeficiency Syndrome (HIV/ AIDS) (PLWHA) worldwide [1, 2]. It can occur throughout the course of HIV [3, 4]. It enhances progression of HIV infection to Acquired Immunodeficiency Syndrome (AIDS) by inducing immune activation [1]. HIV also alters the pathogenesis of TB which greatly increases the risk of developing disease, reactivation of latent TB and leads to more frequent extra pulmonary involvement and atypical radiographic manifestations [1, 3, 4]. PLWHA are 26 times more likely to develop TB than those who are HIV-negative [2]. The global burden of death and disease caused by TB is concentrated particularly in low-income countries [5]. An increased incidence of TB is found mostly in Africa and Asia, where the highest prevalence of co-infection with HIV and TB also occurs [6, 7]. In 2014, globally an estimated 1.2 million (12%) of the 9.6 million people who developed TB worldwide were HIV-positive. About 74% of them were found in African region [5]. HIV-associated TB deaths accounted for 25% of all TB deaths. In the African Region, HIV-positive TB patients were almost twice as likely to die compared with HIV-negative TB patients (9.8% versus 5.1%) [2]. Ethiopia ranks seventh among the world's 22 countries with a high tuberculosis and 27 high MDR TB burden countries in the world [2, 5]. TB is ranked fourth among leading causes of hospital admission and second in causes of hospital death in Ethiopia [5]. In 2013, TB-HIV co-infection rate was 10% in Ethiopia [2].

The World Health Organization has implemented the Standardized Directly Observed Treatment, Short Course (DOTS)/Stop TB Strategy to scale up TB prevention and control which has set a target level of 85% treatment success [8, 9]. But the target was not achieved in all parts of the world. HIV has been accused of being responsible for the non-achievement of target level of treatment success by WHO [8-11]. Globally, the treatment success rate was worse for HIV-positive TB patients (73%) compared with HIV-negative TB patients (88%). While in Africa, the treatment success rate was 75% for HIV-positive TB patients and 84% for HIV-negative [12]. Various factors including poverty, malnutrition, overcrowded living condition, multidrug resistant (MDR) TB and HIV/AIDS have been known to increase the risk of developing the disease and for the continued threat of TB in the world. One of the most important problems in TB prevention and control program is delay in detection and treatment of patients with active TB [9, 11, 13]. Without treatment, TB mortality rates are high. Early detection and proper management of TB are very significant in reducing burdens and impacts of TB in the community and especially among people living with HIV (14, 15). There were varying reports on the treatment outcome of TB-HIV co-infected patients on DOTSs from different parts of the world [9, 13] and Ethiopia [14]. However, treatment outcome of TB and HIV co infected patients were not assessed in Eastern Ethiopia. Therefore, this study aimed to assess treatment outcome of TB among HIV positive patients attending in three hospitals of Eastern Ethiopia.

## Methods

**Study area and population**: Harar town is the capital city of Harari People Regional state, which is one of the most historical towns, located in the eastern part of Ethiopia. It is found at 525 kilometers east of Addis Ababa, the capital city Ethiopia. Based on the 2007 Census conducted by the Central Statistical Agency of Ethiopia (CSA), the town has population of 203,438. It has 19 kebeles [15]. Dire Dawa town is the capital city of Dire Dawa city administrative council. It is a commercial and industrial center located at 515 kilometers from Addis Ababa to the east. Based on the 2007 Census conducted by the Central Statistical Agency of Ethiopia (CSA), Dire Dawa has a population of 341,834 [15]. This study was conducted in antiretroviral treatment (ART) and TB units of two hospitals in Harar town (Harar Defense hospital and Hiwot Fana Specialized University hospital) and one hospital of Dire Dawa Town (Dilchora referral Hospital). All TB-HIV co infected patients registered at the ART and TB units of the above three hospitals who were on TB treatment and their treatment outcome was correctly recorded on their card, were included in the study. The three hospitals were selected based on their high patient load of TB-HIV co infection and the presence of ART and TB units.

**Study design, period and sample size**: Institutional based retrospective cross sectional were used. Clinical records of all 627 TB-HIV co infected patients registered from January 2008 to January 2014 were reviewed from December 2015 to February 2016.

**Data collection method**: The clinical records of the study participants were reviewed by four nurses and two senior nurse supervisors using standard report format of National Tuberculosis and Leprosy Control Programme (NTLCP) [16] for data collection. Information like patients' age, sex, residence, type of tuberculosis and treatment outcome were collected from the clinical record. The clinical cards with missing values were excluded.

**Definitions of terms**: Clinical case and treatment outcome definitions were used according to the standard definitions of National Tuberculosis, and Leprosy Control Programme (NTLCP) [16] and WHO guide lines [17,18]:

**Smear-positive pulmonary tuberculosis (SPPTB)**: A patient with at least two sputum samples positive for AFB microscopy, or a patient who has only one sputum sample positive for AFB microscopy and chest radiographic abnormalities consistent with active pulmonary TB.

**Smear-negative pulmonary tuberculosis (SNPTB)**: Symptomatic illness in a patient with at least two sputum smear examinations negative for AFB on different occasions in whom pulmonary tuberculosis is later confirmed by culture, biopsy or other investigations.

**Extra pulmonary TB (EPTB)**: Tuberculosis of organs other than lungs.

**Other (O)**: A patient who does not fit in any of the above-mentioned categories (e.g. PTB smear negative/EPTB who returns after treatment interruption).

**Chronic (C)**: A TB patient who remains smear-positive after completing a retreatment regimen.

**Treatment outcomes**: Treatment outcomes were divided into six categories according to NTLCP guidelines [16]: A) **Cured**: finished treatment with negative bacteriology result at the end of treatment or sputum smear negative on two occasions at the end of treatment. B) **Completed treatment**: documented treatment completion, but no sputum smear microscopy available at the end of treatment. C) **Defaulted treatment**: a patient who has been on treatment for at least 4 weeks and whose treatment was interrupted for 8 or more consecutive weeks. D) **Died**: a patient who dies for any reason during the course of anti-TB treatment. E) **Transferred out**: a patient who has started treatment and has been transferred to another health facility/clinician (recording and reporting unit) and for whom the treatment outcome is not known at the time of evaluation of treatment results. F) **Successfully treated**: a patient who was cured and/or completed treatment or sum of cases that were cured and completed treatment.

**Data entry and analysis**: The collected data were double entered, cleaned and analyzed by using Statistical Package for Social Sciences (SPSS) software Version 16. Percentage, frequency and other measurements were calculated. Chi square tests were performed to compare treatment outcomes with different study participants' variables. Bivariate and multivariable logistic regression analyses were performed to explore independent variables which were predictors of TB treatment of outcome. The criterion for significance was set with P < 0.05 at 95% confidence interval (CI).

**Ethical consideration**: Ethical clearance was obtained from Haramaya University, College of Health and Medical Science; Institutional Health Research Ethics Review Committee. The patients' clinical records were reviewed anonymously and information obtained during this study was kept confidential.

## Results

**Socio demographic characteristics of study participants**: In this study a total of 627 study participants' clinical records were reviewed. Out of 627 study participants, 57.7% were males. The mean age of participants was 32.52 years (SD ±9.81). Majority of study participants were in the age group of 30-39 years (39.2%) and 19-29 years (34.9%). Almost all (99.0%) of the study participants were urban dwellers. Majority of study participants were Pulmonary TB (PTB) (69.1%) and new (90.7%) patients. Of the total study participants, 42.1% were pulmonary smear negative TB (Table 1).

**Table 1:** Characteristics of HIV seropositive TB patients (n = 627) in three Hospitals in Eastern Ethiopia, 2016

Characteristics			Frequency
			Number (%)
Gender	Male		362(57.7)
	Female		265(42.3)
Age	19-29		219(34.9)
	30-39		246(39.2)
	40-49		105(16.8)
	≥50		34(5.4)
Resident	Urban		621(99)
	Rural		6(1)
Type of TB	PTB	SPPTB	169(27.0)
		SNPTB	264(42.1)
			433/627 =69.1
	EPTB		194(30.9)
Type of TB patient	New		569(90.7)
	Retreatment		36(5.7)
	Defaulter		3(0.6)
	Others*		19(3)

* other = chronic TB cases, Others TB cases, Transfer in TB cases

**Treatment outcomes and success rate**: A total of 17.9% (112/627),60.4%(379/627), 8.6%(54/627) ,0.6% (4/627), 1.8%(11/627) and 10.7(67/627) of the TB-HIV co-infected study participants were cured ,treatment completed, died, failure, defaulter and transferred out respectively. Those participants who were females, in age group 19-29 and rural dwellers were more likely to be in the cured group. Participants in the age group < 18 years, rural dwellers and EPTB were more likely to be in the treatment completed group. Male and those in the age group ≥50 years were more likely to be in the died group. Those who were male, in the age group 40-49 years, urban and in the new TB patients were more likely to be in the treatment failure group. Study participants male, in the age group 19-29 and urban dwellers were more defaulters (Table 2). About 78.3 %( 491/627) and 21.7% (136/627) of the study participants had successful and un successful treatment outcomes respectively. The treatment success was significantly higher among the age groups of < 18 years old, EPTB, new cases and on ART (p< 0.05) (Table 3). Among those participants with unsuccessful treatment outcome, 49.3% (67/136) were transferred out, 39.7% (54/136) were died, 8% (11/136) were defaulter and 2.9% (4/136) were failure. Among transferred out patients, 67.2% and 32.7% were PTB and EPTB, respectively.

**Table 2:** Treatment outcome of TB in HIV seropositive patients (n = 627) by socio-demographic and tuberculosis type in three Hospitals in Eastern Ethiopia, 2016 TB treatment outcome

Characteristics		Cured N (%)	Treatment Completed N(%)	Died N (%)	Failure N (%)	Defaulter N (%)	Transfer out N (%)
Gender	Male	57(15.7%)	219(60.5%)	33(9.1%)	4(1.1%)	7(1.9%)	42(11.6%)
	Female	55(20.8%)	160(60.4%)	21(7.9%)	-	4(1.5%)	25(9.4%)
	Total	112(17.9 %)	379(60.4%)	54(8.6%)	4(0.6%)	11(1.8%)	67(10.7%)
Age	≤ 18	3(13.0%)	19(82.6%)	-	-	-	1(4.3%)
	19-29	55(25.1%)	117(53.4%)	12(5.5%)	1(0.5%)	5(2.3%)	29(13.2%)
	30-39	47(19.1%)	144(58.5%)	26(10.6%)	2(0.8%)	4(1.6%)	23(9.3%)
	40-49	7(6.7%)	74(70.5%)	10(9.5%)	1(1.0%)	-	13(12.4%)
	≥ 50	-	25(73.5%)	6(17.6%)	-	2(5.9%)	1(2.9%)
Residence	Urban	110(17.7%)	375(60.4%)	54(8.7%)	4(0.6%)	11(1.8%)	67(10.8%)
	Rural	2(33.3%)	4(66.7%)	-	-	-	-
Type of TB	PTB	112(26.0%)	221(51.0%)	43(9.9%)	4(0.9%)	8(1.8%)	45(10.4%)
	EPTB	-	158(81.5%)	11(5.7%)	-	3(1.5%)	22(11.3%)
Type of TB patient	New	97(17.0%)	355(62.4%)	47(8.3%)	4(0.7%)	9(1.6%)	57(10.0%)
	Retreatment	13(36.1%)	9(25.0%)	5(13.9%)	-	1(2.8%)	8(22.2%)
	Defaulter	2(66.7%)	-	-	-	1(33.3 %)	-
	Others*	-	15(78.9%)	2(10.5%)	-	-	2(10.5%)

* other = chronic TB cases, Others TB cases, Transfer in TB cases

**Table 3:** Association between different factors, which may affect treatment outcome of tuberculosis among HIV seropositive patients (n = 627) in three Hospitals in Eastern Ethiopia, 2016

Charcteristics		Treatment outcome		
		Successful treatment = 491 N (%)	Un-Successful treatment = 136 N (%)	p-value
Gender	Male	276(76.2%)	86(23.8%)	0.024
	Female	215(81.1%)	50(18.9%)	
Age	<18	22(95.7%)	1(4.3%)	0.010
	20-29	172(78.5%)	47 (21.5%)	
	30-39	191(77.6%)	55(22.4%)	
	40-49	81(77.1%)	24(22.9%)	
	≥50	25(73.5%)	9(26.5%)	
Types of TB	PTB	333(76.9%)	100(23.1%)	0.036
	EPTB	158(81.4%)	36(18.6%)	
ART type	Naive/preART	65 (79.3%)	17(20.7%)	0.013
	on ART	426(78.2%)	119(21.8%)	

**Factors associated with treatment success**: In bivariate analysis age group, ART type and TB type were factors that showed statistically significant association with successful treatment outcome of TB. And also in the final multivariate logistic model age group, ART and TB type were factors associated with successful treatment outcome of TB: age groups of less than or equals to 18 years old participants were about 2 times (adjusted Odds Ratio (AOR) =1.990, 95%CI: 1.01, 3.350) more likely to have higher successful treatment outcome compared to participants with age group of greater than 18 years old. EPTB patients were more than 2 times more likely to (AOR =1.51, 95% CI: 1.12, 3.42) have higher successful treatment outcome when compared to PTB patients. Regarding to ART type: participants on ART were about 2 times (AOR= 1.54, 95%CI= 1.252, 3.910) more likely to have higher successful treatment outcome compared to patients on Naïve/pre-ART. Gender and place of residence did not show any statistically significant association with successful treatment outcome in the Bivariate and Multivariate analysis (Table 4).

**Table 4:** Bivariate and Multivariate analysis of different factors associated with treatment success of TB among HIV seropositive patients in three Hospitals in Eastern Ethiopia, 2016

Characteristics		Treatment outcome		Crude OR	95% Confidence Interval		p-value	Adjusted OR	95% Confidence Interval		p-value
		Successful treatment = 491 N (%)	Un-Successful treatment = 136 N (%)		Lower Bound	Upper Bound			Lower Bound	Upper Bound	
Gender	Male	276(76.2%)	86(23.8%)	1			0.124	1			0.07
	Female	215(81.1%)	50(18.9%)	1.020	0.436	1.988		1.012	0.167	1.914	
Age	<18	22(95.7%)	1(4.3%)	1			0.001	1			0.002
	20-29	172(78.5%)	47 (21.5%)	1.712	1.010	3.987		1.990	1.01	3.350	
	30-39	191(77.6%)	55(22.4%)	1.879	1.001	4.720		1.992	1.02	3.349	
	40-49	81(77.1%)	24(22.9%)	1.901	1.100	3.817		1.996	1.01	3.351	
	≥50	25(73.5%)	9(26.5%)	2.103	1.945	5.909		1.998	1.02	3.354	
Types of TB	EPTB	158(81.4%)	36(18.6%)	1			0.036	1			0.004
	PTB	333(76.9%)	100(23.1%)	2.40	1.821	4.991		1.51	1.12	3.42	
ART type	On ART	65 (79.3%)	17(20.7%)	1			0.013	1			0.023
	Naive/pre-ART	426(78.2%)	119(21.8%)	1.941	1.061	3.101		1.54	1.252	3.910	

EPTB: Extra pulmonary TB; PTB: Pulmonary TB

## Discussion

In this study the treatment outcome of tuberculosis in HIV positive patients treated under DOTS program in the three hospitals of Eastern Ethiopia was 78.3%. This finding was lower than with the national Tuberculosis treatment success rates of 2013 (89 %) [2] and the rate recommended by WHO [11]. It was higher than reports from different parts of Ethiopia; Gondar University Teaching Hospital [19], Felege Hiwot Referral Hospital [20] and public hospitals of eastern and southern zone of Tigray region [21]. The possible explanations for the observed difference of these study findings might be due to high transfer out in the studies of Gondar University hospital [19] and Felege Hiwot Referral Hospital [20]. It might also be due to high death in the studies of Gondar University hospital [19] and public hospitals of eastern and southern zone of Tigray region [21]. The other reason for difference might be difference in patients TB treatment adherence and study period in which the service might be different by time. But, the finding of this study was lower than different studies report from Ethiopia; Enfraz health center [22], Addis Ababa [23], Kolla Diba Health Center [24] and report from Tertiary Care Hospital and ART Centre in Mumbai, India [13]. The possible difference might be due to high transfer out [13, 22-24] and majority of study participants were not co infected with TB-HIV [22-24] in the above studies. Other studies also revealed that HIV patients were more likely to develop TB in the course of the disease [2, 6] and their treatment success was lower [2]. In this study participants in the age groups of greater than 18 years old and Naïve/pre-ART were signicantly associated with lower success rate of treatment outcome. This is similar to studies conducted in Ethiopia [19-21, 23, 24].

In the present study the number of smear negative pulmonary tuberculosis cases (42.1%) relatively higher compared to smear positive and extra pulmonary tuberculosis cases. This was similar to Gondar University hospital [19] and Addis Ababa [23], Ethiopia studies. This might be since HIV positive patients might not expectorate good quality of sputum. Thus, result of their sputum examination can be negative [13, 16]. In this study the PTB patients' success rate were relatively lower than EPTB patients. This might be due to the higher transferred out (67.2%) from PTB patients than EPTB patients (32.7%). The other reason might be HIV- TB co infected patients were less likely to respond to treatment in a similar manner to HIV negative TB. The death rate of this study was 8.6% which is similar to the report from Felege Hiwot Referral Hospital, Ethiopia [20]. But it was higher than reports from other Ethiopian studies; Enfraz health center [22], Addis Ababa [23], Kolla Diba Health Center [24]. The above studies were conducted in a rural health center [22, 24] where bed admission facility was lower and hospital [23] which might have difference with quality of patients' care compared with present study. The present study was conducted in urban hospitals which might hospitalized more critical TB patients. The other reason might be due to the difference of study participants in which the previous three Ethiopian studies [22-24] were conducted in TB patients (both HIV positive and negative) and the study period. In addition, other study also reported HIV-positive TB patients were more likely to die than HIV-negative TB patients [2]. But, the finding of this study was lower than the globally death report of HIV-positive TB patients (11%) and the death report of HIV-positive TB patients (9.8%) in African Region [5], and report from Gondar University Hospital [19]. This might be due to difference in the strength of the TB-HIV control and prevention program currently. The defaulter rate in this study (1.8%) was lower than in other studies conducted in the Public hospitals of eastern and southern zone of Tigray region (2%) [22], Felege Hiwot Referral Hospital (2.5%) [20], Addis Ababa (5.1%) [23] and Gondar University Hospital (18.3%) [20]. The difference might be due to difference in study time, defaulter tracing mechanism and quality of TB-HIV services.

**Limitations of the study**: This study collected information from secondary data which cannot be assessed for different factors identified by different studies as assumed to have impact on treatment outcome of TB/HIV co-infected patients. In addition, some cards with missing values were excluded from this study which might under estimate or overestimate the treatment success. So, attention should be given at time interpretation of this study finding.

## Conclusion

The treatment success rate of TB in TB and HIV co infected patients was 78.3%. Those patients in the age group less than 18, EPTB and on ART had higher treatment success rate. However, it was not signicantly affected by gender and place of residence. In general, the rate of treatment success in this study was lower than recommended by WHO. Therefore, all concerned bodies should plan and implement important activities to improve treatment success rates like enhancing supervision and monitoring TB treatment program in each health institution and improving counseling during the intensive and continuation phases of treatment to enhance TB treatment adherence. The other provision of home visits, motivation of patients, improving defaulter tracing and health information dissemination to reduce treatment interruption. In addition, further large scale prospective study should be conducted to assess the magnitude and different factors affecting the treatment outcome of TB patients in different study sites.

### What is known about this topic

- HIV and TB co infection is one of the major public health problems;
- HIV patients are more likely to develop TB during their life;
- The treatment success rate is worse for HIV-positive TB patients compared with HIV-negative TB patients.

### What this study adds

- Magnitude of treatment outcome of HIV/TB co infected individuals in the region;
- Associated with treatment outcome of HIV/TB co infected individuals in the region;
- The treatment success rate of patients on ART in the region.

## Competing interests

The authors declare no competing interests.
